# HBV Genotyping in HBsAg-Positive Blood Donors from Southwestern Iran

**Published:** 2010-06-01

**Authors:** Mojtaba Oraki Kohshour, Hamid Galehdari, Ali Mohammad Foroughmand, Behnaz Andashti, Mohammad Ali Jalalifar, Ali Bidmeshkipour

**Affiliations:** 1 Department of Immunology, School of Medicine, Ahvaz Jundishapur University of Medical Sciences, Ahvaz, Iran; 2 Department of Genetics, Faculty of Science, Chamran University of Ahvaz, Ahvaz, Iran; 3 Iranian Blood Transfusion Organization Research Center, Ahvaz, Iran; 4 Department of Biology, Faculty of Science, Razi University, Kermanshah, Iran

Dear Editor

In most Asian countries, with a general carrier rate of 5%–35%, hepatitis B virus (HBV) infection is hyperendemic [1]. HBV is a member of Hepadnaviridae, a family of enveloped hepatotropic DNA viruses. It has a circular, partially doublestranded DNA of 3200 nt [2]. This virus can cause severe liver disease with eventual progression to cirrhosis and primary hepatocellular carcinoma (HCC) [3]. HBV possesses a high genetic variability which gives rise to the well-recognized subtypes and genotypes of the virus. In addition, many virus variants are arisen during replication, as a result of nucleotide misincorporation, for lack of any proof-reading mechanisms in the viral polymerase [4]. Therefore, genotyping of HBV is important in determining the route and pathogenesis of the virus. Particular attention has been paid to the differences between the HBV genotypes. It has become increasingly evident that the heterogeneity in the global distribution of HBV genotypes and their sub-genotypes may account for differences in the prevalence of mutations in various populations, the clinical outcomes of HBV infections, and the response to antiviral therapy. There are several methods for HBV genotyping. For instance, sequencing of entire virus genome is one of the most accurate but time-consuming and expensive methods. To solve this problem, other techniques were developed; those included multiplex nested polymerase chain reaction (PCR) that was first described by Naito et al., using six pairs of type specific primers [3], which was used in the present study to detect the HBV genotypes. A total of 66 HBsAg-positive blood donors were included in this study. HBV genotypes were determined by multiplex nested PCR with six pairs of HBV genotype-specific primers (A to F) and direct sequencing of PCR products. Our finding indicated that the genotype D is the predominant genotype in southwestern Iran, as has been reported in other studies from Iran and other Mediterranean countries [5]. To validate the obtained results from multiplex nested PCR, some randomly selected PCR products were subjected to the direct sequencing. Regarding the sequence alignment by the NCBI-blast program (www.ncbi.org), all the sequenced samples showed high homology to the genotype D. Our study also indicated that the predominant genotype with HBsAg blood donors in southwestern Iran is similar to those of the other Mediterranean areas.
